# Transmission pathways of *campylobacter* spp. at broiler farms and their environment in Brandenburg, Germany

**DOI:** 10.3389/fmicb.2022.982693

**Published:** 2022-10-06

**Authors:** Benjamin Reichelt, Vanessa Szott, Lennard Epping, Torsten Semmler, Roswitha Merle, Uwe Roesler, Anika Friese

**Affiliations:** ^1^Department of Veterinary Medicine, Institute for Animal Hygiene and Environmental Health, Freie Universität Berlin, Berlin, Germany; ^2^Genome Sequencing and Genomic Epidemiology, Robert Koch Institute, Berlin, Germany; ^3^Department of Veterinary Medicine, Institute for Veterinary Epidemiology and Biostatistics, Freie Universität Berlin, Berlin, Germany

**Keywords:** campylobacter, environment, transmission, broiler, WGS

## Abstract

Broiler meat is widely known as an important source of foodborne *Campylobacter jejuni and Campylobacter coli* infections in humans. In this study, we thoroughly investigated transmission pathways that may contribute to possible *Campylobacter* contamination inside and outside broiler houses. For this purpose we carried out a comprehensive longitudinal sampling approach, using a semi-quantitative cultivation method to identify and quantify transmissions and reservoirs of *Campylobacter* spp.. Three german broiler farms in Brandenburg and their surrounding areas were intensively sampled, from April 2018 until September 2020. Consecutive fattening cycles and intervening downtimes after cleaning and disinfection were systematically sampled in summer and winter. To display the potential phylogeny of barn and environmental isolates, whole genome sequencing (WGS) and bioinformatic analyses were performed. Results obtained in this study showed very high *Campylobacter* prevalence in 51/76 pooled feces (67.1%) and 49/76 boot swabs (64.5%). Average counts between 6.4 to 8.36 log_10_MPN/g were detected in pooled feces. In addition, levels of 4.7 and 4.1 log_10_MPN/g were detected in boot swabs and litter, respectively. Samples from the barn interior showed mean *Campyloacter* values in swabs from drinkers 2.6 log_10_MPN/g, walls 2.0 log_10_MPN/g, troughs 1.7 log_10_MPN/g, boards 1.6 log_10_MPN/g, ventilations 0.9 log_10_MPN/g and 0.7 log_10_MPN/g for air samples. However, *Campylobacter* was detected only in 7/456 (1.5%) of the environmental samples (water bodies, puddles or water-filled wheel tracks; average of 0.6 log_10_MPN/g). Furthermore, WGS showed recurring *Campylobacter* genotypes over several consecutive fattening periods, indicating that *Campylobacter* genotypes persist in the environment during downtime periods. However, after cleaning and disinfection of the barns, we were unable to identify potential sources in the broiler houses. Interestingly, alternating *Campylobacter* genotypes were observed after each fattening period, also indicating sources of contamination from the wider environment outside the farm. Therefore, the results of this study suggest that a potential risk of *Campylobacter* transmission may originate from present environmental sources (litter and water reservoirs). However, the sources of *Campylobacter* transmission may vary depending on the operation and farm environmental conditions.

## Introduction

*Campylobacter* spp. remains an important cause of concern in broiler production as it is the most frequently reported food-borne pathogen in the European Union (EU). In 2019, there were 220,682 confirmed cases of human campylobacteriosis. Poultry meat is considered the most important source of human infection (Meldrum et al., 2005; Stafford et al., 2008; Facciola et al., 2017; Vetchapitak and Misawa, 2019). *Campylobacter* spp. enters the food chain through poultry colonization primarily at farm level but may also occur through secondary contamination at market and consumer levels. Therefore, the elimination or at least reduction in the poultry reservoir must be considered a key step to successfully combat the bacterium in the food chain (Lin, 2009). The epidemiology of *Campylobacter* in commercial broiler production is not yet fully understood. The prevalence of *Campylobacter* spp.-positive poultry broiler flocks varies by region, season and production system (Bahrndorff et al., 2013; Murphy et al., 2018)*. C. jejuni* and *C. coli* are the main causes of campylobacteriosis in humans (Kaakoush et al., 2015). Several studies have shown that both *C. jejuni* and *C. coli* infections in humans occur more often in summer than in other seasons (Nielsen et al., 2013; Bessède et al., 2014). The distinct seasonal pattern of *Campylobacter* emergence in broiler farms suggests that seasonal changes in the environment may play an important role among other contributing factors affecting *Campylobacter* spp. survival and spread. A variety of sources have been identified and *Campylobacter* spp. has been shown to spread rapidly within the flock after its introduction (Koolman et al., 2014). Nevertheless, the exact time window of *Campylobacter* occurrence and transmission in broiler farms before and after detection in broiler chickens is not yet fully known. Farm control strategies such as well-implemented hygiene protocols have shown to reduce the incidence of *Campylobacter* (Gibbens et al., 2001; Borck Hog et al., 2016). It has been suggested that environmental contamination during rearing period may be responsible for colonization of subsequent broiler flocks. Possible risk factors for *Campylobacter* colonization are poorly implemented biosecurity and practices as well as the age of broilers, presence of other livestock animals within a 1 km radius, ventilation systems (insects), number of barns, employees and farm equipment, seasonality and thinning procedure (Gibbens et al., 2001; Hald et al., 2004, 2008; Nicholson et al., 2005; Stern, 2008; Horrocks et al., 2009; Newell et al., 2011; Patriarchi et al., 2011; Ridley et al., 2011; Wagenaar et al., 2013; Carron et al., 2018; Murphy et al., 2018). Feces of animals and wild birds, spreading of farmyard manure, compost, and domestic animals such as dogs and cats have previously been described as environmental reservoirs for *Campylobacter* spp. (Whiley et al., 2013). Water bodies are considered as possible *Campylobacter* reservoirs (Jones, 2001; Cools et al., 2003; Pitkanen, 2013). *Campylobacter* control on farms is a cumbersome task as the pathogen may survive and persist in a variety of environments and hosts (Lin, 2009; Silva et al., 2011). Preventing the entry or onset of *Campylobacter* into broiler farms, it is of great importance to determine potential *Campylobacter* transmission routes as well as relevant environmental reservoirs at broiler farms. The scope of this study was to ascertain *Campylobacter* transmissions at conventional broiler farms. Therefore, we investigated three different broiler farms in Brandenburg over 3 years. Specifically, we combined a semi-quantitative approach to estimate contamination levels as well as whole-genome sequencing, as a source-tracking tool. The discriminatory power of the whole-genome approach was used to provide further insight into possible unidirectional transmission pathways.

## Materials and methods

### Broiler farms and rearing

In total, three broiler chicken farms (A, B, C; features explained in Table 1) in Brandenburg were studied between May 2018 and September 2020 (Table 2). *Campylobacter* spp. presence was determined by pooling 10 individual fecal or cecal droppings. At each farm, four barns were individually examined in two consecutive fattening cycles in summer and winter.

**Table 1:** Farm characteristics and broiler rearing conditions in Brandenburg, Germany, 2018–2020.

	Farm A	Farm C	Farm B
Farm area	1 ha	3.4 ha	0.84 ha
Rearing capacity	126,000	348,000	74,000
Barns	4	15	4
Breed used	Ross 308	Ross 308 and Cobb 500	Ross 308, (Hubbard)^a^
Stocking density	39 kg/m^2^		39 kg/m^2^; (25 kg/m2)^a^
Rearing system	Raised on the ground floor with fresh litter, (feed and water) *ad libitum*, all in all out		
Rearing period	36 to 42 days		36 to 42 (60 days)^a^
Diet	Starter, grower and finisher		
Outdoor access	Not provided		
Thinning	Day 35		Day 35, (53)^a^
Wastewater collection (sludge/slurry pits)	Open	Underground	Open
Distance to livestock/processing units	/	Nearby poultry slaughterhouse in the immediate vicinity (0.5 km)	Personnel and material traffic with a dairy farm (2 km)
Distance to waterbodies	Water ditches (<0.5 km) Lake (1.3 km)	Lakes (1.5–1.7 km)	Water ditches (< 0.5 km) Lake (0.5 km)
Distance to other farms investigated in this study	30 km	5 km	5 km

a Change to alternative broiler management to accommodate slow-growing breeds towards the beginning of 2020.

**Table 2:** Comprehensive overview of all broiler farm visits in Brandeburg, Germany, 2018–2020.

Farm A	Farm B	Farm C
**Date**	**Visit**	**E**	**B**	**Date**	**Visit**	**E**	**B**	**Date**	**Visit**	**E**	**B**
04.18	A_S18_sc1	N	8/8	05.18	B_S18_sc1	N	3/18	05.18	C_S18_sc1	N	0/30
06.18	A_S18_1	1/24	17/40	06.18	B_S18_1	0/24	20/40	06.18	C_S18_sc2	N	0/30
06.18	A_S18_C&D	N	0/30	07.18	B_S18_C&D	N	0/30				
07.18	A_S18_2	0/24	23/40	08.18	B_S18_2	0/24	23/40				
01.18	A_W18_1	0/26	0/40	01.18	B_W18_1	1/25	13/40	02.19	C_S18_sc3	N	0/30
01.18	A_W18_C&D	N	0/28	02.18	B_W18_C&D	N	0/41	02.19	C_S18_sc4	N	0/30
03.18	A_W18_2	1/26	0/40	03.18	B_W18_2	3/26	20/40				
07.19	A_S19_1	0/24	5/40	04.18	B_W18_3^C^	N	14/20								
07.19	B_S19_1	0/24	9/40	08.19	C_S19_sc5	N	4/30
a	a	11.19	C_S19_sc6	N	2/30
12.19	A_W19_1	0/24	12/24	12.19	B_W19_1	0/24	0/40	11.19	C_W19_1	0/24	9/40
12.19	A_W19_C&D	N	0/36	12.19	B_W19_C&D	N	0/36	11.19	C_W19_C&D	N	0/36
				02.19	B_W19_2	0/24	0/36	02.19	C_W19_2	0/24	0/40
				02.20	B_S20_1	0/22	12/40	07.19	C_S20_1	1/22	23/40
				07.20	B_S20_C&D	N	0/36	07.19	C_S20_C&D	N	0/36
a	09.20	B_S20_2	0/21	11/40	08.19	C_S20_2	0/24	12/40

E, environment; B, Barn; N, not investigated;

W, winter; S, summer; sc, screening; C&D, cleaning and disinfection; C, cow. ^a^suspended investigation.

### Biosecurity

Biosecurity measures can be described as follows: at farm A and B, the presence of standard personal hygiene, as well as disinfectant footpads or baths, were available. Farm C, however, applied additional measures. This included strict hygiene practices in the anterooms, which were also cleaned and disinfected frequently during fattening. Furthermore, rubber boots were changed at the entrance of the anteroom by dipping the boots used on the farm premises in disinfectant baths to enter the anteroom and then changing into a separate pair of boots for exclusive use inside each respective barn. In addition, the boots used within the barns were cleaned and disinfected on a regular basis. Furthermore, a wheel washing disinfection basin at the entrance of the farm premises was available. In addition, a commercially available hydrogen peroxide-based product was continuously added to the drinking water system at Farm C. At all farms, bedding was removed immediately after each rearing period. Premises and barns were cleaned and disinfected and then remained empty for about 2 weeks. Cleaning and disinfection (C&D) of the broiler barns was carried out by the farm personnel in farms A and C and by an external service provider at farm B. C&D consisted of dry cleaning, followed by wet-cleaning and disinfection with commercial disinfectants selected by broiler manufacturers approved by the German Veterinary Society (DVG) for animal husbandries. Procedures were performed as suggested by German Agricultural Society (DLG). Apart from the hygiene practices mentioned above, farm C was the only farm that used feed and drinking water additives containing bulk elements (calcium and magnesium), trace elements (selenium), vitamins (E and D3), amino acids (lysin, threonin, methionin, tryptophan) and organic acids (propionic acid, lactic acid, sorbic acid, L-ascorbic acid, acetic acid, formic acid and its salt ammonium format and citric acid). In addition, farm C nebulized various essential oils (peppermint-, eucalyptus-, and menthol oil). However, these additional measures were withdrawn in the third quarter of 2019 following the transfer to a new operator (farm management change).

### Sampling design

Once *Campylobacter* spp. was detected on a farm, a predefined, farm-specific sampling scheme was applied according to the geographic location of the buildings and exposure to potential environmental influences, such as wind direction and orientation of access roads and vehicle and personnel traffic. Briefly, four barns were investigated twice at the end of two consecutive fattening periods (rearing 1 and rearing 2) in summer (S) and winter (W) on each broiler farm. In between, the houses were also sampled after depopulation, removal of the litter and C&D. However, the start of the visits on the different farms varied (Table 1). Since farm C remained *Campylobacter* negative in several screenings, the sampling scheme described above could not be implemented until August 2019. Each sampling was conducted after thinning and at least 1 week prior to complete removal of the flock. Per farm visit, four barns were investigated and each of them was sampled as follows: three fecal matter and associated samples (FMAS), which were pooled feces, litter, boot swabs as well as seven samples from the barn interior (air, dust, swabs from drinker nipples and trays, troughs, wall, fan, physical entrance barrier (board; see detailed description in the section below). In addition, we collected six of the following samples each (air, boot swabs, gauze swabs, and water) yielding a total of 24 environmental samples. It is to note that seasonal influences sometimes prevented obtaining the same water samples. To determine *Campylobacter* existence after C&D, six gauze swabs (from drinker nipples and trays, troughs, wall, wall-to-floor transition, fans, entrance board and floor) were collected at each barn. Additionally, boot swabs, water from the drinking water supply as well as chicken litter residues were sampled. Deviating from this regular scheme, 20 additional fecal samples from dairy cows at the affiliated dairy farm of farm B were included (B_W18_3).

### Sample collection

*Pooled feces and litter samples* consisted of the material from 10 individual feces or cecal droppings. Pooled litter samples were each collected from 10 different locations. To be as uniform as possible, samples were collected in the same manner whenever possible: feces samples (mixture of cecal dropping and feces), and litter (dry and wet at 5 to 10 m intervals) at the same areas. After C&D, if present, samples of litter residue, often mixed with cleaning and disinfecting agents were scraped from cracks and cavities and pooled. Furthermore, 10 g of each cow patty from farm B was examined as an individual sample. Samples were collected in sterile 120 ml specimen containers with spatulas (VWR, Radnor, Pennsylvania).

*Boot swabs* inside the barn were taken by walking the entire length of the barn and returning on the opposite side (Ridley et al., 2011). Environmental boot swabs from the outside were collected by walking the entire length of the chicken barn at a distance of 1 m from the barn, including areas with grass, concrete, soil, or puddles (Bull et al., 2006).

*Gauze Swabs* were previously moistened with 5 ml Nutrient Broth No. 2 (NB) and used to swab an area of 10 × 10 cm. Samples were collected from the barn interior inside the barns. Therefore, fans, drinker nipples and trays, troughs, the walls of the barn and the wooden board at the entrance were swabbed as described previously (Bull et al., 2006; Ridley et al., 2011). Environmental gauze swabs were taken in the same manner from farm equipment (tractors, carts, wheelbarrows, buckets,) and exposed surfaces close to the emission source around or on near barn ventilation fans.

*Air samples* were collected using the Coriolis® MICRO microbial air sampling device (Bertin Technologies, Montigny Le Bretonneux, France). Sampling in the barn interior was performed for 1 min at a flow rate of 250 L of air/min at four previously determined standard sampling points (i.e., total volume of 1,000 L) to gain one pooled sample per barn, in total four per rearing cycle (Ahmed et al., 2013). In the environment, however, the Coriolis was positioned at ascending distances within the exhaust air stream of the facility, considering the overall wind direction. For this purpose, the parameter of the Coriolis was set to 4 min at a flow rate of 250 L air/min (i.e., total volume of 1 m^3^) per sampling point. In total 6 air samples were taken. The instrument collects particles from the air in a fluid stream. Subsequently, airborne particles were collected in Coriolis® μ cones filled with NB. The device is capable of saturating particles with a pore diameter of 0.5–50 μm, as specified by the manufacturer. Dust was scraped from various surfaces from the barn interior in sterile 120 ml specimen containers.

*Water* from the environment such as surface water from ditches adjacent to the farm or within a 0.5 km range of the farm site and water bodies further away (<1 km) was collected in 500 ml sterile multi-purpose containers (Sarstedt, Sarstedtstraße 1, 51,588 Nümbrecht Germany). Water present at the farm premises such as puddles, residues in wheel tracks and waterholes, was sampled in a sterile screw cap tube, 50 ml (Sarstedt, Sarstedtstraße 1, 51,588 Nümbrecht Germany), by submerging, wearing sterile gloves. Wastewater from storage pits, basins, drains, and gullies was skimmed using a telescoping pole and a. All water samples were stored under chill conditions and examined within 2 h after sampling. After C&D, drinking water was drained from the supply system into sterile 500-ml multipurpose containers.

### Laboratory processing of samples

#### Isolation and quantification of *campylobacter* spp.

Samples were prepared for semi-quantitative analysis according to ISO/TS 10272-3:2010 (method for the semi-quantitative determination of *Campylobacter* spp.). Therefore, all samples were diluted 1:8 in Preston Broth [PB; NB supplemented with Preston *Campylobacter* selective supplement (SR0117; Oxoid, Wesel, Germany), growth supplement (SR0232; Oxoid, Wesel, Germany) and defibrinated horse blood (SR0050; Oxoid, Wesel, Germany)]. Boot swabs, on the other hand, were diluted in 100 ml in PB while gauze swabs were transferred to 20 ml PB. For semi-quantitative analysis, 10 g pooled feces and litter and 5 g dust were diluted in PB. All samples were then homogenized at 200 rpm for 2 min using a laboratory Smasher (bioMérieux, Durham, United States). Air samples collected in Coriolis® μ cones (Berlin Technologies) were thoroughly vortexed using a vortex shaker (VWR, Darmstadt, Germany) and diluted in PB. For water sample preparation, samples were, if necessary, divided into aliquots and centrifuged at 16000 rpm for 10 min. Subsequently, the pellets were diluted in PB. All samples previously prepared at a ratio of 1:8 were then diluted 10-fold in PB. All dilutions were then incubated for 24 h at 37°C under microaerophilic conditions (85% nitrogen, 10% carbon dioxide, 5% oxygen) and afterward streaked out on quartered modified cefoperazone deoxycholate agar (mCCDA; CM0739; Oxoid, Wesel, Germany) supplemented with CCDA selective supplement (SR0155; Oxoid, Wesel, Germany) using 10-μl inoculation loops (Sarstedt, Nürnberg, Germany). Plates were then incubated for 48 h under the same conditions. Subsequently, all dilutions were counted semiquantitatively, and the highest dilution with confirmed *Campylobacter* growth was used to determine the MPN (Most Probable Number) using a modified MPN table according to ISO/TS 10272-3:2010/Cor.1:2011(E). Putative colonies were isolated and streaked out on Columbia blood agar (ColbA) with 5% sheep blood. Putative colonies on ColbA plates were then incubated as described before. Afterward, colonies were analyzed using a Bruker Microflex ® system for matrix-assisted laser desorption ionization time-of-flight mass spectrometry (MALDI-TOF MS) as previously described (Golz et al., 2020). *Campylobacter* isolates that were species confirmed with “high-confidence identification” score ≥2.00 (Bessède et al., 2011; Emele et al., 2019; Hsieh et al., 2019) were grown overnight in 3 ml PB and 1.5 ml of bacterial solution was then stored in glycerol stocks at −80°C applying 0.5 ml 50% glycerol for further analysis.

#### Whole-genome sequencing

For WGS analysis, a total of 113 MALDI–TOF MS confirmed *Campylobacter* isolates (99 *C. jejuni* and 14 *C. coli*) from FMAS and barn interior (1 isolate each per barn) as well as the environment (1–3 isolates per sample) were systematically selected. For DNA extraction, *Campylobacter* strains stored at −80°C were cultured on ColbA at 42°C under microaerophilic conditions for 24 h. After subculture for 18 ± 2 h, cells were suspended in phosphate-buffered saline PBS (Dulbecco A) pH 7.3 ± 0.2 (BR0014G, Oxoid limited, Basingstoke Hampshire, England) at OD_600_ = 0.2, corresponding to ~9 log_10_ cell counts per ml (Krüger et al., 2014). The cell suspension was centrifuged (16,000 g, 5 min) and afterward Genomic DNA was extracted using the GeneJET Genomic DNA Purification Kit (Thermo Fisher Scientific). Hereafter, the quality of the DNA was tested spectrophotometrically using NanoDrop Thermo Fisher Scientific. In addition, a Thermo Fisher Scientific Qubit (Qubit 2.0 Fluorometer) was used to measure the DNA yield. Bacterial DNA was sequenced using the Illumina NextSeq 550 platform with 2×150 bp (Illumina Inc. San Diego, CA). Raw reads were treated for quality control, trimmed for adapters and genomes were *de novo* assembled using SPAdes v3.12 (Bankevich et al., 2012) with the careful option. Thereafter, an in-house database (Golz et al., 2020) was used for gene annotation with Prokka v.1.14 (Seemann, 2014) and subsequently used as input for Roary v3.12.070 (Page et al., 2015) to calculate the pan-genome size and core genome alignment with 95% sequence identity. Maximum likelihood Phylogenetic trees based on the core genome alignment for *C. coli* and *C. jejuni* isolates were built with RAxML v.8.2.1071. Subsequently, the phylogenetic tree was visualized with meta data using Phandango (Hadfield et al., 2017). Finally, iTOL (Letunic and Bork, 2021) was employed to create a visualization of the C. *jejuni* pangenome phylogeny with meta and MLST data. Furthermore, core genome MLST (cgMLST) analysis was performed by Ridom Seqsphere+ v. 6.0.0 (2019–04; Ridom, Muenster, Germany) using the cgMLST scheme of 1,343 gene targets previously proposed (Cody et al., 2017). The phylogenetic trees were visualized with GrapTree v.1.5.0 (Zhou et al., 2018). A minimum spanning tree (MST) based on the cgMST profiles were calculated with NINJA NJ (neighbor-joining; Wheeler, 2009). The BLAST-based tool “mlst”^1^ based on the *Campylobacter jejuni/coli* database of pubmlst.org was used to obtain MLST profiles which were subsequently used to carry out an *in silico* analysis of the previously described seven housekeeping genes (aspA, glnA, gltA, glyA, pgm, tkt, uncA; Dingle et al., 2001). New multilocus sequence typing (MLST) alleles and MLST-ST types were uploaded to PubMLST.org/campylobacter.

### Statistical analysis

All quantitative data were compiled in a Microsoft Excel spreadsheet. Analysis of prevalence and distribution frequency of bacterial isolates from broiler flocks and their environment were performed using IBM SPSS Statistics for Windows, version 27 (IBM Corp., Armonk, N.Y., United States). Data were analyzed using generalized linear mixed models (GLMMs), with farm visits as a random factor. One model was a logistic approach modeling the probability of *Campylobacter* presence as a dependent variable, the second model was linear for log_10_MPN/g values and included only positive samples. Year, season, sampling type and the farm were used as dependent factors. Posthoc pairwise comparisons were adjusted using the least significant difference method (LSD; logistic model) or Bonferroni correction (linear model). Odds Ratios (OR) including 95%- confidence intervals (CI) were calculated for the logistic model. value of ps *p* < 0.05 were regarded as statistically significant. Graphs were created using GraphPad Prism 9 (2020) GraphPad Software 2,365 Northside Dr. Suite 560 San Diego, CA 92108.

## Results

### *Campylobacter* spp. prevalence at broiler farms

*Campylobacter* spp. was isolated from farms A and B immediately after the first visit (screening) in April and May 2018. Farm C, however, remained *Campylobacter* spp. negative over four screenings until August 2019 (Table 1). A total of 19 rearing cycles were examined on three different broiler farms (A, B, C). Across all samples, *Campylobacter* spp. prevalence was 17.8%. Of these, *C. jejuni* was the most commonly MALDI-TOF MS identified species (93.5%) but *C. coli* (4.8%) was occasionally isolated, exclusively from farm B (Table 3). Furthermore, in some rearing cycles mixed cultures were found (1.9%). Sorted by category, *Campylobacter* spp. was found in 136/228 FMAS (59.6%), in 73/532 samles from the barn interior (13.7%) and in 7/456 environmenal samples (1.5%) and 14/20 (70%) from dairy cattle feces at farm B. After C&D, *Campylobacter* was not cultured from any of the samples (*n* = 309; Table 3). Within FMAS, pooled feces (67.1%) and boot swabs (64.5%) showed the highest *Campylobacter* prevalence. In the mixed logistic regression model, the prevalence of FMAS was significantly higher than in environmental samples (*p* < 0.001, OR 458, 95% CI 178–1,175). Among barn interior samples, prevalence varied between 0% (dust) and 26.3% (drinker nipples and trays; Table 3). Even in the barn interior, the chance for positive samples was 12 times higher than in the farm environment (*p* < 0.001, OR 12.3, 95% CI 5.5–27.5). *Campylobacter* spp. was detected in 4.4% of water samples, 0.9% each in boot and surface swabs and never in air samples. Comparing the summer and winter months, *Campylobacter* spp. was 22 times more frequently detectable in summer (*p* = 0.021, OR = 21.7, 95% CI 1.6–297.4; Table 4). Comparing the individual prevalence of all samples, there were no significant differences between the years (*p* > 0.05). Moreover, the comparison of the three individual farms considering all samples showed no statistically significant difference in *Campylobacter* spp. prevalence (*p* > 0.05; Table 4).

**Table 3:** Summary of total samples (concentrations, prevalence, and *Campylobacter* species type) collected at broiler farms in Brandenburg, Germany, 2018 to 2020.

*Category and sample*		*Campylobacter* prevalence all visits		*Campylobacter* concentrations all visits (Log_10_MPN/g)			No. (%) positive for			*Campylobacter* prevalence	
										Summer		Winter		Farm A		Farm B		Farm C	
		No. positive/ total collected	Total % positive	Average	Min	Max	*C. jejuni*	*C. coli*	*C. jejuni /coli Mix*	No. positive/ total collect	Total % positive	No. positive/ total collected	Total % positive	No. positive/ total collected	Total % positive	No. positive/ total collected	Total % positive	No. positive/ total collected	Total % positive
FMAS^1^		136/228	59.6	5.2	0.36	8.36	125 (91.9)	10 (7.4)	1 (0.7)	95/120	79.2	41/108	38	39/72	54.2	76/108	70.4	21/48	43.8
Pooled feces	51/76	67.1	6.4	2.36	8.36	46 (90.2)	4 (7.8)	1 (2.0)	36/40	90	15/36	41.7	16/24	66.7	26/36	72.2	9/16	56.3
Boot swabs	49/76	64.5	4.7	2.36	7.36	47 (95.9)	2 (4.1)	–	34/40	85	15/36	41.7	14/24	58.3	26/36	72.2	9/16	56.3
Litter	36/76	47.4	4.1	0.36	7.36	32 (88.9)	4 (11.1)	–	25/40	62.5	11/36	30.6	9/24	37.5	24/36	66.7	3/16	18.8
Barn interior		73/532	13.7	1.8	0.36	4.36	71 (97.3)	1 (1.4)	1 (1.4)	60/280	21.4	13/252	5.2	28/168	16.7	32/252	12.7	13/112	11.6
Air	8/76	10.5	0.7	0.36	1.36	8 (100)	–	–	6/40	15	2/36	5.6	3/24	12.5	5/36	13.9	–	–
Dust	0/76	–	–	–	–	–	–	–	0/40	–	0/36	–	–	–	–	–	–	–
Drinker	20/76	26.3	2.6	0.36	4.36	20	–	–	17/40	42.5	3/36	8.3	9/24	37.5	8/36	22.2	3/16	18.8
Troughs	14/76	18.4	1.7	1.36	2.36	14	–	–	12/40	30	2/36	5.6	4/24	16.7	6/36	16.7	4/16	25
Ventilation	4/76	5.3	0.9	0.36	2.36	4	–	–	4/40	10	0/36		2/24	8.3	–	–	2/16	12.5
Wall	9/76	11.8	2.0	0.36	4.36	9	–	–	7/40	17.5	2/36	5.6	5/24	20.8	4/36	11.1	–	–
Board	18/76	23.7	1.6	0.36	4.36	17 (94.4)	–	1 (5.6)	14/40	35	4/36	11.1	5/24	20.8	9/36	25	4/16	25
Environment		7/456	1.5	0.6	0.36	1.36	5 (71.4)	–	2 (28.6)	2/233	0.9	5/223	2.2	2/148	1.4	4/214	1.9	1/94	1.1
Air	0/114					–	–	–	0/60	–	0/54	–	–	–	–	–	–	–
Water	5/114	4.4	0.6	0.36	1.36	4 (80)	–	1 (20)	2/53	3.8	3/61	4.9	2/40	5.0	2/52	3.8	1/22	4.5
Boot swabs	1/114	0.9	1.4	1.36	1.36	1 (100)	–	–	0/60	–	1/54	1.9	–	–	1/54	1.9	–	
Gauze swabs	1/114	0.9	0.4	0.36	0.36	1 (100)	–	–	0/60	–	1/54	1.9	–	–	1/54	1.9	–	
Additional investigations																									
Cow feces	14/20	70%	2.6	1.36	3.36	10 (71.4)	4 (28.6)								14/20	70%		

1 Fecal matter and associated samples.

**Table 4:** Estimated regression coefficients, *t* and *p* values, Odds-Ratio and corresponding 95% confidence intervals (CI) of the mixed logistic and linear regression models. The random effect is visits within farm, the fixed effects are year, season, category and farm. Dependent variables are the probability for positive samples for the logistic and log_10_MPN/g for the linear model. Rows without OR or *t*-value represent the reference group. In this line, the variable’s global *p* values are given.

	Mixed logistic regression model					Mixed linear regression model				
	Regression coefficient	Value of *p*	OR^a^	95% confidence interval		Regression coefficient	*t*-value	Value of *p*	95% confidence interval	
				Lower limit	Upper limit				Lower limit	Upper limit
Constant	−8.193	<0.001	0.000	0.000	0.012	0.385	0.441	0.664	−1.430	2.200
2018	2.086	0.275	8.052	0.190	340.597	0.441	0.670	0.521	−1.072	1.955
2019	0.372	0.843	1.451	0.036	58.157	−0.806	−1.117	0.293	−2.437	0.824
2020	0.000	0.363	.	.	.	0.000	.	0.131	.	.
Summer	3.075	0.021	21.660	1.578	297.380	−0.354	−0.679	0.516	−1.550	0.841
Winter	0.000	.	.	.	.	0.000	.	.	.	.
FMAS^b^	6.127	<0.001	457.980	178.480	1175.178	5.024	8.383	<0.001	3.842	6.206
Barn interior	2.509	<0.001	12.298	5.498	27.508	1.399	2.306	0.022	0.203	2.595
Environment	0.000	<0.001	.	.	.	0.000	.	<0.001	.	.
Farm A	−0.461	0.809	0.630	0.015	26.844	0.222	0.295	0.775	−1.484	1.927
Farm B	0.000	>0.999	1.000	0.040	25.084	−0.141	−0.215	0.835	−1.630	1.347
Farm C	0.000	0.940	.	.	.	0.000	.	0.785	.	.

a Odds-Ratio.

b Fecal matter and associated samples. *p*-values *p* < 0.05 were regarded as statistically significant.

### *Campylobacter* concentrations

**Figure 1:**
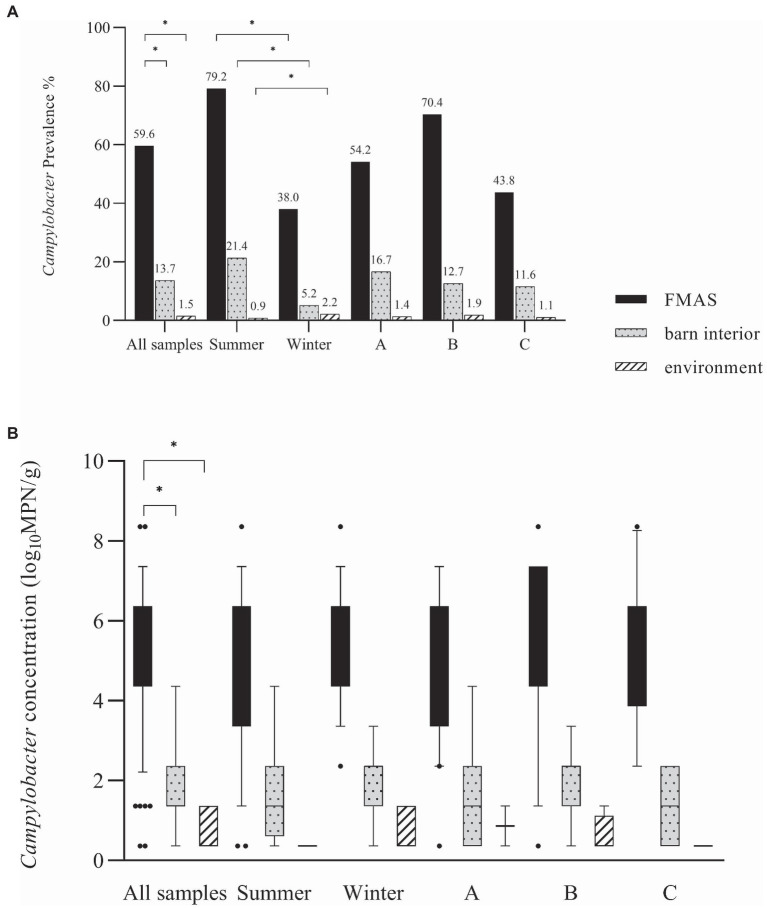
**(A)** *Campylobacter* spp. prevalence in (%) for all samples, seasons and farms. **(B)** Log_10_ most probable number (MPN) of *Campylobacte*r spp. in all samples, seasons and farms. Black bars represent FMAS, grey bars represent the barn interior and dotted bars represent the environment. Bars marked by an asterisk differ significantly (*p* < 0.05). The box plots show the 5th and 95th percentiles (whiskers).

*Campylobacter* spp. concentration in FMAS was high (mean of 5.2 log_10_MPN/g, *n* = 136). Of those, pooled feces showed the highest concentration (mean value 6.4 log_10_MPN/g, *n* = 51) and up to 8.36 log_10_MPN/g (maximum bacterial concentration). In comparison, boot swabs (*n* = 49) and litter (*n* = 36) had significantly (*p* < 0.05) lower bacterial concentration (mean value 4.7 and 4.1 log_10_MPN/g, respectively). The *Campylobacter* load in samples taken from the barn interior differed significantly (*p* < 0.0001) when compared to pooled feces (Bonferroni post-hoc tests, Tables 3, 4). Barn interior samples, gauze swabs from the surface of drinker nipples and trays showed the highest bacterial concentration (mean value 2.6 log_10_MPN/g, *n* = 20), followed by wall (mean value 2.0 log_10_MPN/g, *n* = 9), board (mean value 1.6 log_10_MPN/g, *n* = 18), troughs (mean value 1.7 log_10_MPN/g, *n* = 14) and ventilation (mean value 0.9 log_10_MPN/g, *n* = 4; Table 3). Air samples demonstrated the lowest *Campylobacter* counts (mean value 0.7 log_10_MPN/g, *n* = 8). In contrast, environmental samples (air, water, boot swabs and gauze swabs) showed the lowest *Campylobacter* concentration (average mean value 0.6 log_10_MPN/g, *n* = 7). In detail, *Campylobacter* spp. was cultivated from five different water samples: (i) from a water retention pond on farm A in the first rearing cycle in summer 2018 (A_S18_1), (0.36 log_10_MPN/g), (ii) from an adjacent ditch on farm A in the first rearing cycle in winter 2019 (A_W19_1), (1.36 log_10_MPN/g), (iii) from a puddle next to the barn on farm B (0.36 log_10_ MPN/g) as well as (iv) an adjacent ditch near farm B (similar concentration of 0.36 log_10_MPN/g) each in the second rearing cycle in winter 2019 (B_W19_2) and (v) from a small pool of rainwater in a transport box near the barns on farm C in the first rearing cycle in summer 2020 (C_S20_1), (0.36 log_10_MPN/g; Table 3). In addition, *Campylobacter* spp. was grown from one environmental gauze swab taken from work material (hand trucks) stored in the immediate vicinity of the barns on farm B during the first rearing cycle in winter 2019 (B_W19_1; 0.36 log_10_MPN/g). Likewise, *Campylobacter* spp. was cultivable from a boot swabs taken from wheel tracks containing rainwater and manure residue on farm B during the second rearing cycle in winter 2019 (B_W19_2; 1.36 log_10_MPN/g). The linear regression model showed that only the sampling type significantly influenced the log_10_MPN/g values (*p* < 0.001). Again, FMAS (5.0 log_10_MPN/g) and barn interior samples (1.4 log_10_MPN/g) had significantly higher values than the environment (Tables 3, 4; Figure 1).

### Core and accessory genome analysis

**Figure 2:**
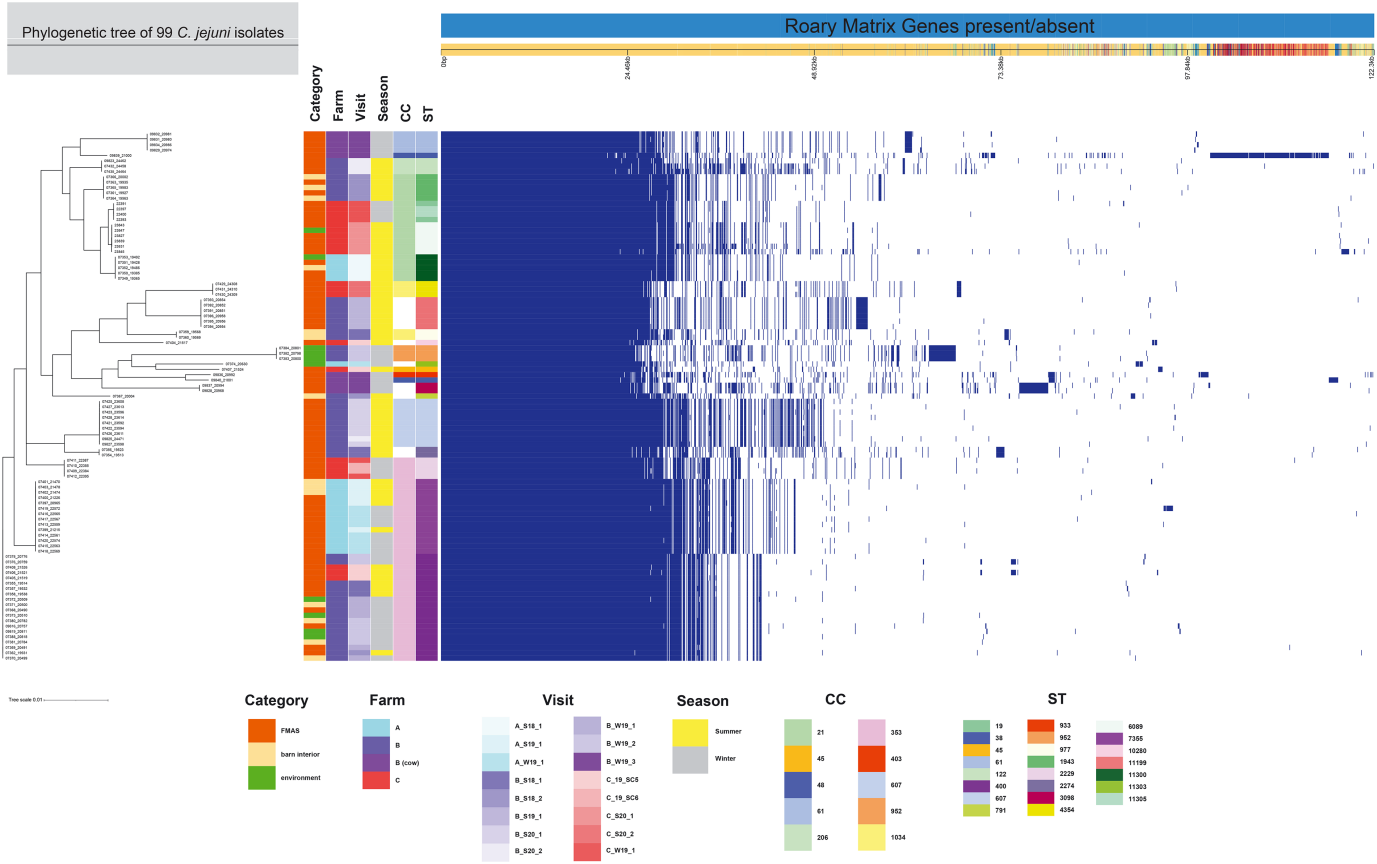
The figure was drawn using the phandango.net web application (Hadfield et al., 2017). The left panel shows the maximum likelihood tree of 99 *C. jejuni* isolates (1,234 genes) based on the alignment of the core-genome calculated with RAxML. The top panel shows a single representative nucleotide sequence (0 bp-122.3 kb); contigs (fragments are colored by similarity) and genes are derived from the pan-genome content. The right panel displays the Roary pangenome sorted from core genes on the left to accessory genes to the right, with presence (blue) or absence (white) of blocks relative to genes and contigs in the pan-genome. The middle features metadata [category, farm, visit, season, Clonal complex (CC), and Sequence type (ST)]. The white blocks represent unassigned CCs and STs.

**Figure 3:**
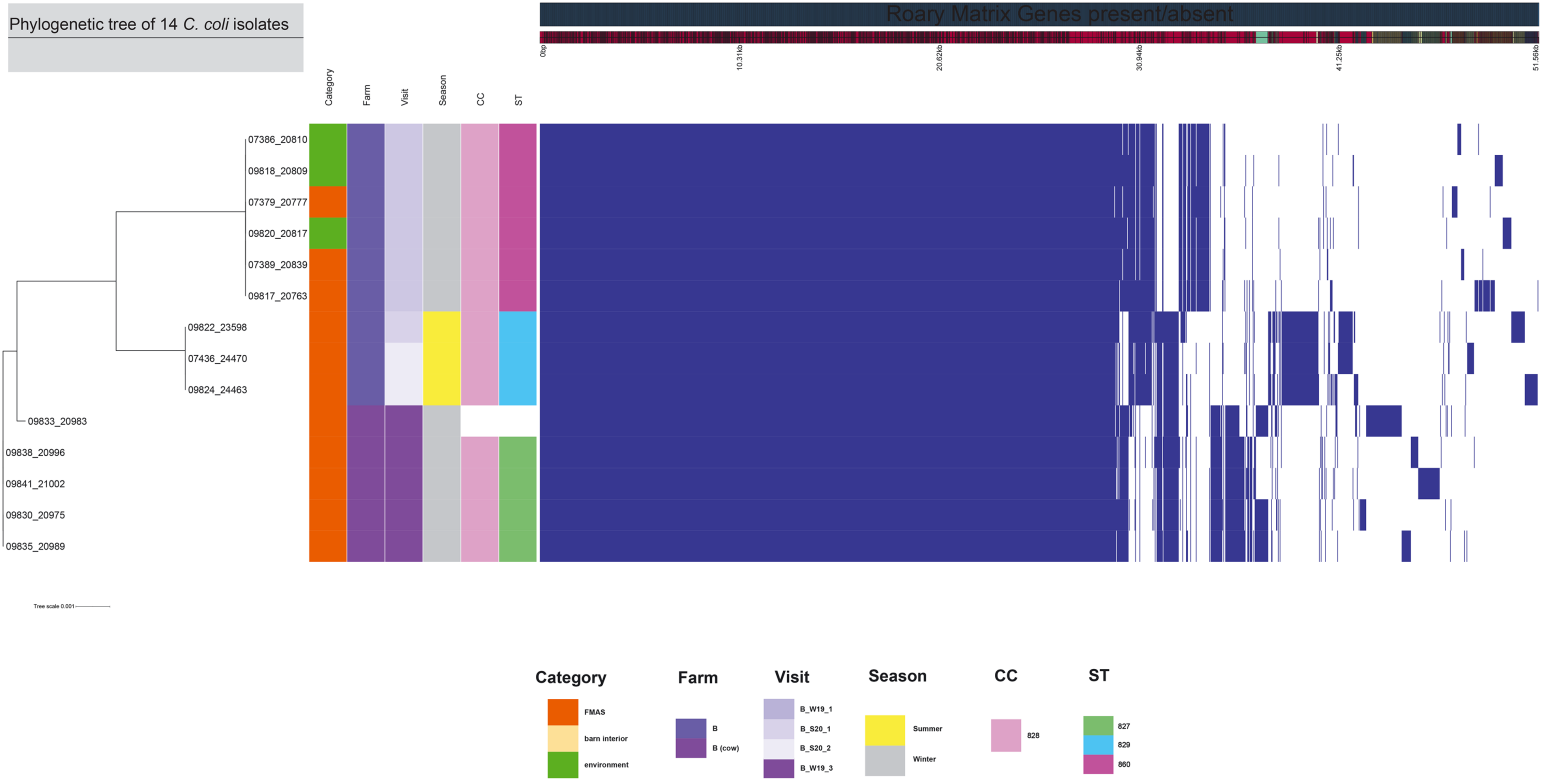
The figure was drawn using the phandango.net web application (Hadfield et al., 2017). The left panel shows the maximum likelihood tree of 14 *C. coli* isolates (1,482 genes) based on the alignment of core-genome calculated with RAxML. The top panel shows a single representative nucleotide sequence (0 bp-51.56 kb); contigs (fragments are colored by similarity) and genes are derived from the pan-genome content. The right panel displays the Roary pangenome sorted from core genes on the left to accessory genes to the right, with presence (blue) or absence (white) of blocks relative to genes and contigs in the pan-genome. The middle features metadata [category, farm, visit, season, clonal complex (CC), and sequence type (ST)]. White blocks represent unassigned CCs and STs.

The 99 *C. jejuni* genomes studied consisted of 6,113 genes, including 1,234 core genes and 88 softcore genes. Their accessory genome consisted of 915 shell genes and 3,876 cloud genes (Figure 2). Based on 95% sequence identity of 14 *C. coli* genomes in this study, a total of 2,576 genes were identified in their core and accessory genomes, of which an estimated 1,482 formed the core genome and 1,094 formed the accessory genome (530 sell and 564 cloud genes; Figure 3).

### Population structure (MLST-types)

**Figure 4:**
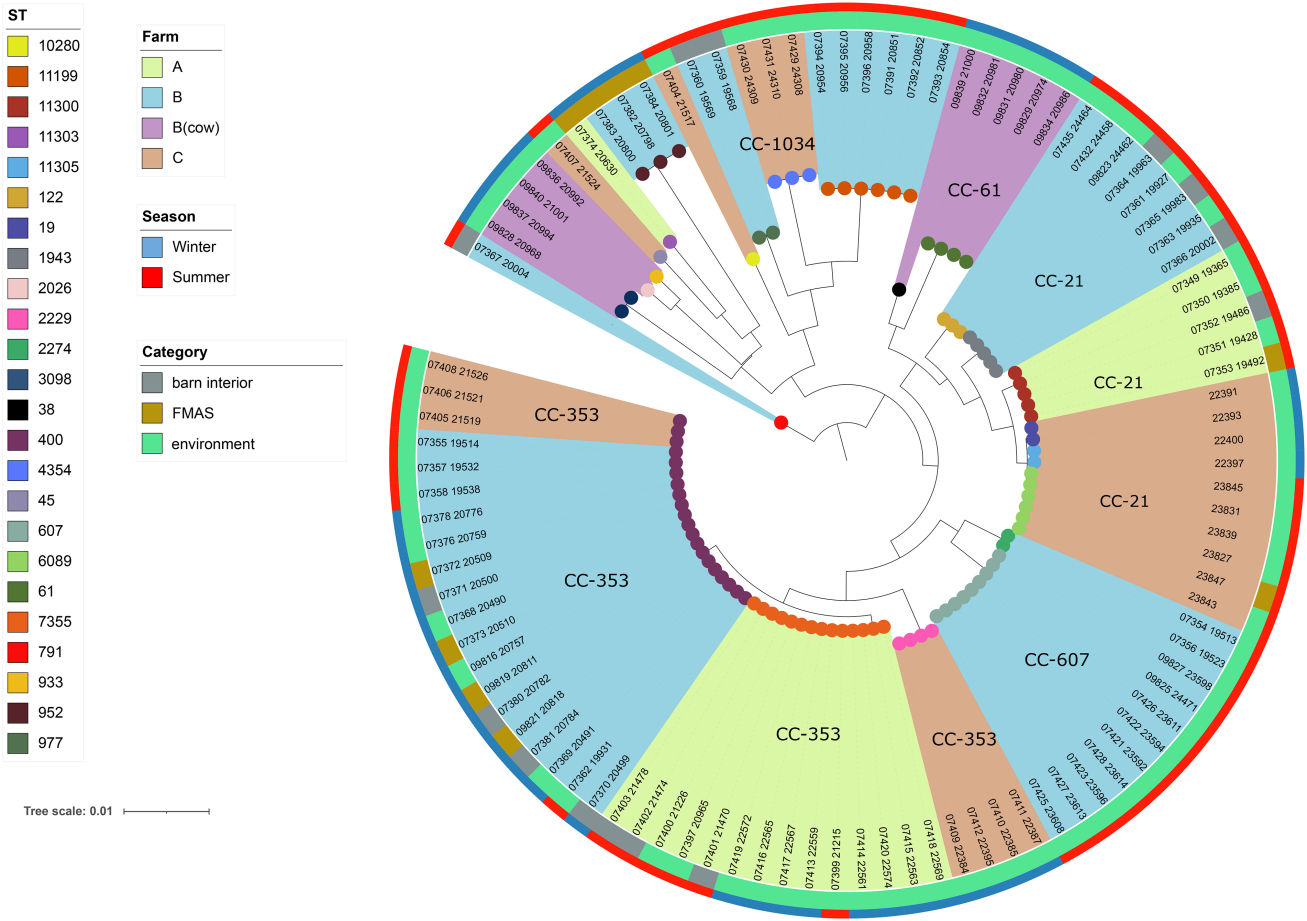
Maximum-likelihood phylogenetic tree based on the alignment of 99 *C. jejuni* core genomes (1,234 genes) using roary v3.12.070 and RAxML. The phylogenetic tree was visualized using iTOL v6 and rooted at the midpoint. Brown, light blue and light green shades display the different broiler farms. Clonal Complexes (CC) are indicated. Sequence types (STs) are displayed at the leaf node. The outer ring represents the season while the inner ring displays isolate category (FMAS, barn interior or environment).

Among the 113 isolates studied [*C. jejuni* (*n* = 99) and *C. coli* (*n* = 14)], a total of 27 different sequence types (STs) which could be categorized into 11 clonal complexes (CC) were allocated using the seven housekeeping genes MLST. CCs are defined as a group of STs which share similarities to a central allelic profile. The most abundant STs identified from *C. jejuni* isolates were ST-400 with 20 members (20.2%) and ST-7355 with 14 members (14.1%), both belonging to CC-353 (Figure 2). Furthermore, the majority of isolates from the three broiler farms (A, B, and C) studied were assigned to CC-353 and 21 whereas a minor fraction was assigned to 607, representing 38, 20 and 9 *C. jejuni* isolates, respectively (Figure 2). However, CC-607 was uniquely observed at broiler farm B and unrelated to any other farm. As mentioned earlier, broiler farm B was the only farm where *C. coli* was detected. With respect to CC categorization, CC-828 was predominant for most of the *C. coli* isolates found at broiler farm B (Figure 4). In comparison, most isolates from broiler farm A were assigned to ST-7355 (CC-353) as well as ST-11300 (CC-21). The latter were simultaneously confirmed in the barn and in the environment at visit A_S18_1. In addition, a new ST (ST-11303) was detected at visit A_W19_1 near the barn in an adjacent ditch. Compared to farm A, farm B demonstrated a broader diversity of different ST types in *C. jejuni* isolates. To be precise, ST-400 from CC 353 was detected alongside ST-1943 from CC-21 and ST-607, ST-122 from CC-206 and ST-977 from CC-1034. Furthermore, two more STs (ST-791 and ST-2274) could not be assigned to a specific CC. Besides, we determined a new type of ST-11199 in *C. jejuni* isolates. At broiler farm B, the dominant and recurrent ST-400 (CC-352) was abundant across multiple fattening periods in 2018 and 2019 (Figure 2). Barn and environmental isolates were assigned to ST 400 in summer 2018 as well as in winter 2019 (B_W19_1/2; Figure 2). Further, three *C. jejuni* isolates from a nearby water body (B_W19_2) were assigned to ST-952. As mentioned before, all *C.coli* isolates were assigned to CC-828. Specifically, isolates from farm B were determined as ST- 829 and ST- 860. ST-829 was observed in consecutive rearing cycles while ST-860 was observed in only 1 cycle (B_W19_2). However, ST-860 could also be detected in the environment (Figures 2, 4). Towards the end of 2019 (C_19_SC5), *C. jejuni* isolates were typed ST-400, ST-45 and ST-10280 at farm C. Subsequently, *C. jejuni* isolates of visit C_19_SC6 were typed ST- 2229 of CC 353. The same ST-2229 was also detected in the following rearing cycle of visit C_W19_1 in the winter months. However, another two *C. jejuni* isolates (ST-19 and ST-11305) belonging to CC-21 were found. In summer 2020, at visit C_S20_1, on the other hand, ST-6089 of CC-21 was detected in *C. jejuni* isolates from FMAS and water from the environment. In the following rearing cycle (C_S20_2), barn isolates from FMAS were assigned to ST-4354 from CC-1034. Additional investigations of the neighboring dairy farm (distance *ca.* 2 km) of farm B revealed *C. coli* isolates which were typed ST-827 (Figure 4). In addition, several *C.jejuni* isolates of ST-3098, ST-61, ST-933, ST-2026 and ST-38 were found.

### Phylogenetic analysis of the *Campylobacter jejuni* and *Campylobacter coli* core genomes

For the core genome phylogenetic analysis, 99 *C. jejuni* genomes (1,234 genes; Figure 2) and 14 *C. coli* genomes (1,482 genes; Figure 3) were used to construct a maximum likelihood phylogenetic tree (ML tree) from the Roary core-genome alignment. Analysis of the ML tree revealed close relationship between strains isolated from FMAS, barn interior and the environment (Figures 2, 3). Furthermore, identical *Campylobacter* types that occurred in consecutive fattening periods over several seasons and between different farms were identified and clustered. Three major *C. jejuni* clusters (ST-7355, ST-400 and ST-607) and a minor cluster (ST-2229) were identified from the ML tree and Coregenome phylogeny paired with metadata (Figures 2, 4). All three clusters consisted of isolates from FMAS, barn interior, and the environment. *C. coli*, on the other hand formed two major clusters, primarily consisting of broiler (FMAS, environment; ST-860 and ST-829) as well as cow isolates (ST-827; Figure 3). However, the core genome phylogeny shows that new distinct *Campylobacter* types emerged during the fattening period and disappeared thereafter (after C&D). In total, 8 minor clusters were formed in the ML tree, always clustering with the same ST types (Figures 2, 3). In contrast to isolates forming clusters, some individual strains with distinct pan-genomes are observed in the center of the ML tree. For example, strain 20630 allocated to ST-11303 and a minor cluster of isolates (20798, 20800 and 20801 of ST-952; Figure 2), show discrepancies in the ML tree phylogeny and their pan-genome (Figure 2). Moreover, strain 21000 isolated from cow feces, allocated to ST-48 and often associated with humans, broilers, and wild birds, shows a large accessory genome which is often associated with niche versatility.

## Discussion

### Prevalence and concentration

The results of this study show frequent detection (59.6%) and high concentrations (6.4 log_10_MPN/g) of *Campylobacter* spp. in pooled feces. In contrast, findings in the vicinity of the farms were sporadic and showed significantly lower concentrations (Table 3). These results are consistent with observations from other studies (Petersen and Wedderkopp, 2001; Shreeve et al., 2002; Zweifel et al., 2008; Schets et al., 2017; Mohammed and Abdel Aziz, 2019; Tang et al., 2020). Intensive sampling of the barn interior showed that drinker nipples and trays, troughs, the physical exit barrier (board) and vents/fans were often contaminated with *Campylobacter*, albeit at low levels. The surfaces of the aforementioned barn equipment are often contaminated with chicken feces and contaminated litter. Detailed molecular typing of isolates isolated from swab samples (barn equipment and air) revealed that *Campylobacter* isolates were identical to those found in FMAS. The latter suggests a rapid distribution of *Campylobacter* spp. within the broiler houses (Hertogs et al., 2021). Contamination of water pipes may occur as a result of a drastic increase in fecal excretion following flock colonization with *Campylobacter*. Indeed, drinking water systems contaminated with feces are often described as potential reservoirs in poultry houses (Frosth et al., 2020). Drinking water treatment with chlorinated water or water enriched with organic acid shows partial effects on *Campylobacter* colonization and transmission as previously observed in part at farm (Newell and Fearnley, 2003; Hutchison et al., 2004; Sahin et al., 2015) Other studies showed that drinkers without cups were less prone to become *Campylobacter* reservoirs (Borck Hog et al., 2016). In this context, troughs filled with contaminated feed can also act as a vehicle for horizontal *Campylobacter* transmission (Hald et al., 2000; Silva et al., 2011). A board (physical exit barrier) contaminated with chicken feces can be another potential *Campylobacter* reservoir, potentially allowing a release into the environment as it separates the barn from the entry area (vestibule; Bull et al., 2006; Agunos et al., 2014; Battersby et al., 2016). A potential release in the environment can take place especially when this barrier is not used properly (lack of sufficient C&D, missing change of footwear). As indicated previously, *Campylobacter* spp. was not found in any of the dust samples, which is probably explained by its sensitivity to desiccation (Fernández et al., 1985; Schets et al., 2017). *Campylobacter* positive swabs from ventilation systems indicate circulating *Campylobacter* bound to airborne particles of fecal matter. In agreement, we were able to cultivate and quantify *Campylobacter* spp. from broiler house air, albeit at very low levels as shown by semi-quantitative analysis (1.4 log_10_MPN/g in 1 m^3^ of house air). These findings are consistent with previous results (Bull et al., 2006; Chinivasagam et al., 2009; O’Mahony et al., 2011; Johannessen et al., 2020). An optimized in barn management of litter (acid treatment, reduced moisture), water (sanitization, additives (Jansen et al., 2014), phages (Wagenaar et al., 2005; Kittler et al., 2013), feed (bacteriocins, essential oils (Szott et al., 2020; Wagle et al., 2020; Allaoua et al., 2022) temperature and humidity control may reduce *Campylobacter* occurrence in which reduce the overall airborne *Campylobacte*r transmission (Sahin et al., 2002, 2015; Newell and Fearnley, 2003). However, in contrast to previous observations (Bull et al., 2006; Ridley et al., 2011), we were unable to detect cultivable *Campylobacter* in air samples outside the barns. A possible explanation for this could be the strong dilution effect in the ambient air, which leads to the detection limit being exceeded. Nevertheless, we found isolates from the air that had the same sequence types as isolates from FMAS, which matched isolates from puddles and water retention ponds. This could be an indicator of possible airborne emissions from farms. *Campylobacte*r spp. was rarely isolated from environmental matrices (water, boot swabs, gauze swabs) at very low concentrations. Previous studies found similar or higher detection rates of *Campylobacte*r spp. in the environment of poultry productions (Bull et al., 2006; Hansson et al., 2007; Ridley et al., 2011; Thakur et al., 2013; Hertogs et al., 2021).

### Seasonal effect

In broilers, a distinct seasonality with prevalence peaks for *Campylobacter* in summer and autumn has been described (Meldrum et al., 2005; McDowell et al., 2008; Hartnack et al., 2009; Whiley et al., 2013; Sahin et al., 2015; Smith et al., 2016; Baali et al., 2020). Accordingly, we found distinct variations in the detection rates and distribution of *Campylobacter* spp. in the sampled farms. To be precise, we found seasonally high *Campylobacter* prevalence in FMAS in summer, while significantly lower rates were detected in winter (Figure 1). Indeed, in the winter months some rearing cycles were even completely negative for *Campylobacter* spp. (2 cycles at farm B, 1 cycle on farm C (Table 3). It is hypothesized that the entry of *Campylobacter* is lower in winter because there is significantly less animated vectorial movement on the farms as a result of an absence of insects and wild birds and rodents (Hald et al., 2007; Jonsson et al., 2012; Djennad et al., 2019). Nevertheless, we have also detected *Campylobacter* spp. in environmental samples during winter investigations. We assume that favorable conditions such as high relative humidity (RH), rainfall, lower UV radiation due to cloudy weather may have contributed to a prolonged environmental survival of *Campylobacter* spp. in winter, as illustrated before (Jones, 2001; Murphy et al., 2006; Whiley et al., 2013; Mulder et al., 2020). Indeed, we detected phylogenetically very similar *Campylobacter* in both, the environment and broiler flocks during the winter (Farm B). It can therefore be surmised that the area surrounding the broiler flocks (environment) served as a reservoir at Farm B.

### Environmental findings

At farm B, WGS revealed phylogenetic identity of *Campylobacter* strains from the environment and the barn. Analysis of boot swabs taken from wheel tracks, water samples and gauze swabs taken from contaminated work material indicates that these specific sites are potential *Campylobacter* transmission pathways into or out of the barn. Since these have already been identified as of particular concern in other studies (Bull et al., 2006; Messens et al., 2009; O’Mahony et al., 2011; Ridley et al., 2011; Zhang et al., 2017), it seems possible that water residues in wheel tracks and puddles, in particular, represent a reservoir for *Campylobacter* spp. on farm B in our study. At farm A, the isolates obtained from a water collection basin and barn samples are genetically identical and typed (ST-11300). Recurrent strains in subsequent fattening periods (ST-7355 from CC-353) were also detected (Figures 3, 4). It seems quite possible that the water collection basin served as a reservoir for these dominant recurrent strains. At farm C, sequence type ST-6089 was detected once in an isolate from a water sample that came from a carelessly placed transport box filled with rainwater. This isolate matched isolates from inside the barn. Sporadically found reservoirs (predominantly water-associated) in the immediate barn environment could cause colonization pressure of certain genotypes from the environment, as already inferred by others (Bull et al., 2006; Ellis-Iversen et al., 2012; Zhang et al., 2017). Besides, *Campylobacter* spp. were isolated at farm A and B in more distant water during winter. However, neither ST types nor pangenome phylogenies matched the farm isolates at any time. The occurrence of *Campylobacter* spp. in surface waters could also be associated with agricultural runoffs in this region (Kemp et al., 2005). Nevertheless, the isolate from an adjacent trench was assigned to ST-11303, which could not be assigned to any other isolate. Interestingly, the environmental isolate from a ditch found near farm B could be assigned to ST-952, which has previously been associated with wild birds, rabbits and environmental waters (Kwan et al., 2008). Although we were unable to establish an epidemiological link between these isolates and isolates from farm A and B, these results nevertheless indicate the persistence of *Campylobacter* spp. in aquatic environments and support their importance as a reservoir beyond the immediate vicinity of poultry farms (Van Dyke et al., 2010; Mughini-Gras et al., 2016; Schets et al., 2017; Mulder et al., 2020). Furthermore, we found isolates of the same sequence type (ST-400 CC-353) on farm B and C, which showed genetic relation by the core genome phylogeny. It can only be speculated, that those isolates were spread from farm B to farm C (distance of 5 km) as that type was never isolated on farm C before (Figures 2, 4).

### Farm features

As opposed to farm A and B, farm C remained *Campylobacter* negative over several visits (from 2018 through the end of 2019; Table 1). Thereafter, *Campylobacter* spp. was sporadically detected towards the end of 2019 and even frequently isolated in the further course of the study at detection rates similar to those of farm A and B. We suspect that the gradual emergence of positive *Campylobacter* broiler flocks is related to changes in farm staff. In particular, changes in flock management, biosecurity, and hygiene practices were observed. In this context, following changes that may have led to *Campylobacter* spp. establishment were observed: i) withdrawal of food and drinking water additives (acidification with organic acids and the nebulization of essential oils and ii) reduction of hygiene practices that previously exceeded the standard used in farm A and B. For example, the elimination of a hygiene protocol that had previously been strictly followed (farm-owned rubber boots, barn-owned rubber boots in the vestibule of each barn, strict disinfection regularly). The former might be related to organic acids, as beneficial effects on flock colonization have been demonstrated in other studies (Ivanov, 2001; Line, 2002; Jansen et al., 2014). Improved hygiene measures appear to delay or prevent the occurrence of *Campylobacter* spp. as previously noted (Humphrey et al., 1993; Gibbens et al., 2001; Pattison, 2001; Shreeve et al., 2002; McDowell et al., 2008; Georgiev et al., 2016). In contrast to farm A and C, farm B had a neighboring dairy farm which was managed by the same personnel. To determine a possible transfer, exchange or pathway between dairy and poultry farming, the dairy farm was included once in our investigation. However, molecular typing assigned different STs (ST-3098, ST-61, ST-933, ST-2026 and ST-38) to *C. jejuni* isolates from dairy cows compared with those isolated from broilers at farm B. With regard to the occurrence of *C. coli* at fam B, all isolates were categorized as CC-828. CC-828 is mainly associated with strains isolated from agricultural and environmental sources (Sheppard et al., 2010). We determined different STs that were present in cattle isolates (ST-827) and others (ST-829, ST-860) only present in chicken pooled feces and environmental isolates of that period. Although these STs belong to the same CC-828, core genome phylogeny analysis shows discrepancies between *C. coli* isolates of the two major clades (Figure 3). However, it is to note that we broaden the investigation on dairy cows only once. Nevertheless, other studies reported transmission of *Campylobacter* genotypes from cattle to broiler (Bull et al., 2006; Ridley et al., 2008, 2011; Zweifel et al., 2008; Ellis-Iversen et al., 2009; Frosth et al., 2020). However, we were not able to determine a transmission of *Campylobacter* spp. between the dairy and broiler farm.

### Reoccurring *campylobacter* strains and respective reservoirs

Comparing isolates that were frequently detected in multiple consecutive fattening periods at farm A and B, molecular typing and phylogenetic analysis showed high genetic relatedness. It is possible that *Campylobacter* isolates either survived in the respective broiler houses after cleaning and disinfection (Cardinale et al., 2004; Huneau-Salaün et al., 2007) or originated from reservoirs outside the broiler houses. At farm A, for example, we observed a reoccurring strain of ST-7355 in two consecutive seasons. Other researchers also demonstrated sporadic re-emergence of identical *Campylobacter* strains in subsequent fattening periods (Petersen and Wedderkopp, 2001; Shreeve et al., 2002; Johnsen et al., 2006; Ridley et al., 2011; Ellis-Iversen et al., 2012). Intensive sampling of the barn interior after cleaning and disinfection between two consecutive fattening periods, however, revealed no culturable *Campylobacter*. Possibly we were not able to verify *Campylobacter* spp. presence, as the bacterium transitioned into a viable but not culturable (VBNC) state. Based on our observations, it might be feasible that *Campylobacter* was accumulated into the environment through contaminated litter and thus persisted in sporadic reservoirs (puddles, wheel tracks) in the vicinity of the barn. Although we were able to identify temporary reservoirs contaminated with *Campylobacter*, we were unable to identify them as the definitive source of colonization of broiler flocks as we were unable to detect *Campylobacter* in the same reservoir in consecutive fattening periods. Whether this is due to detection limits or VBNC formation is unclear. The latter remains to be explored in further studies.

## Conclusion

The results of this study show that despite a systematic approach, it has proven difficult to identify *Campylobacter* transmissions and definitive sources of broiler colonization. This study highlights that *Campylobacter* transmissions *via* contaminated litter may play an important role in the formation of *Campylobacter* reservoirs, for example, in puddles or wheel tracks in the environment of broiler farms. Conversely, we also found sporadic *Campylobacter* in water bodies surrounding broiler farms A and B (distance >2 km). Contaminated water bodies may generally serve as a potential source of infection for wild animals, which may then colonize adjacent broiler flocks by acting as horizontal vectors. Besides, our observations suggest that the sources and pathways of *Campylobacter* transmission may vary considerably between different broiler farms in terms of the type of operation, the microclimate, the type of ventilation, hygiene management and the surrounding environmental conditions (distance to water bodies or other farms). Therefore, the aforementioned measures need to be targeted to reduce the risk of new or recurrent *Campylobacter* genotypes potentially circulating and colonizing newly introduced broiler flocks.

## Data availability statement

The datasets presented in this study can be found in online repositories. The names of the repository/repositories and accession number(s) can be found in the article/Supplementary material.

## Author contributions

BR performed the experiments, collected, analyzed, and interpreted the data, and drafted the manuscript and figures, with critical evaluation and support of all other authors. VS helped with the experiments and lab work as well as drafting the manuscript. LE and TS performed processing and downstream analyses of all sequencing data. RM performed the statistical analysis of the data provided. AF designed the study, as well as critically revised the manuscript. All authors contributed to the article and approved the submitted version.

## Funding

This work was financially supported by the Federal Ministry of Education and Research (BMBF) within the framework of the consortium “PAC-Campy” (IP1/01KI1725A).

## Footnotes

^1^ https://github.com/tseemann/mlst
