# Supplementary material for: Transmission pathways of campylobacter spp. at broiler farms and their environment in Brandenburg, Germany
Source: Front Microbiol. 2022 Oct 6;13:982693. doi: 10.3389/fmicb.2022.982693 (PMC9598865; doi:10.3389/fmicb.2022.982693)
Supplement: Supplementary file 1 [file Data_Sheet_1.docx]

**Supplementary Material:** Transmission pathways of *Campylobacter* spp. on broiler farms and their environment in Germany

Benjamin Reichelt^1*^, Vanessa Szott^1^, Lennard Epping^2^, Torsten Semmler^2^, Roswitha Merle^3^, Uwe Roesler^1^ and Anika Friese^1^

**MLST Trees and Distribution**

**A)**


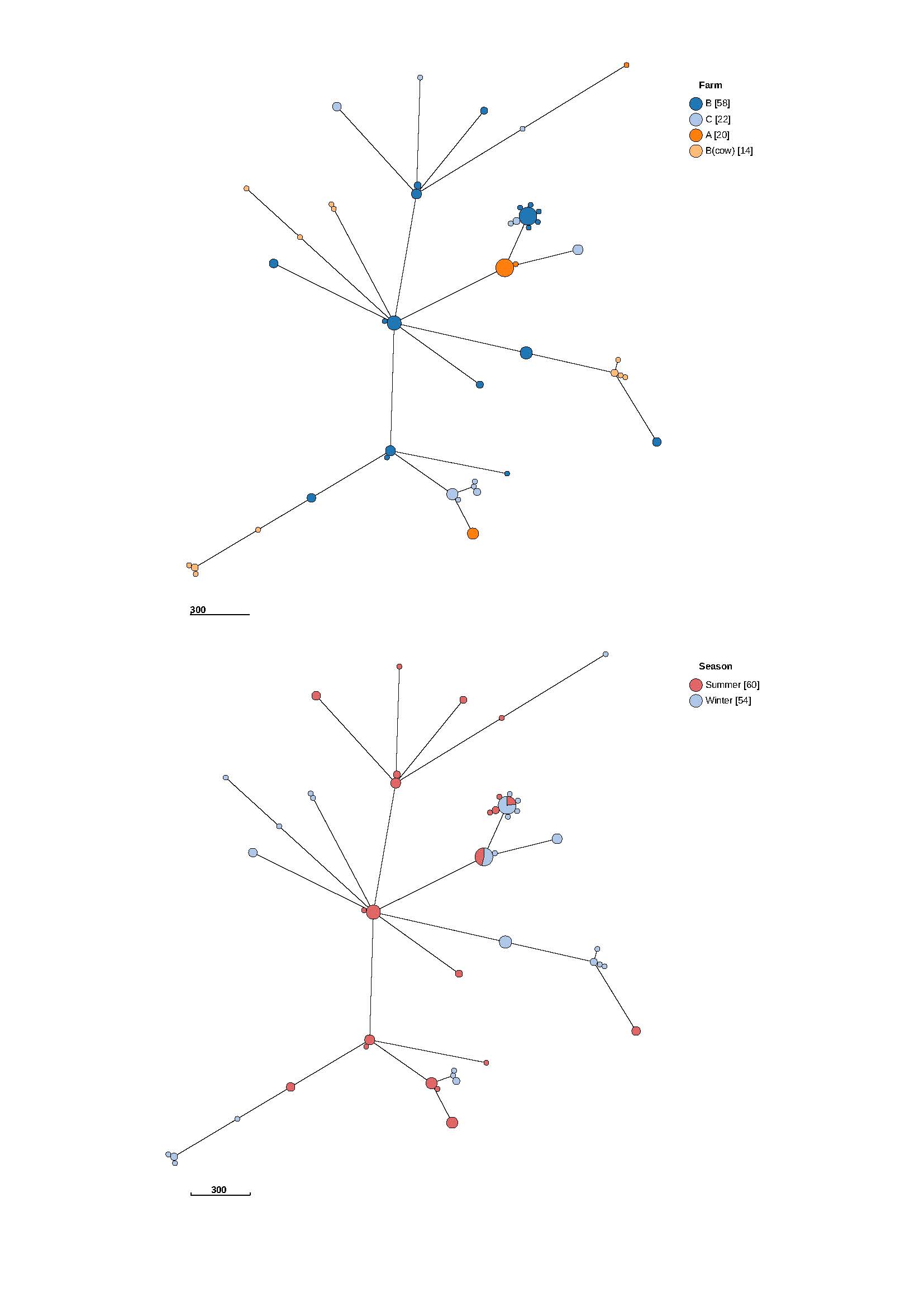


**B)**


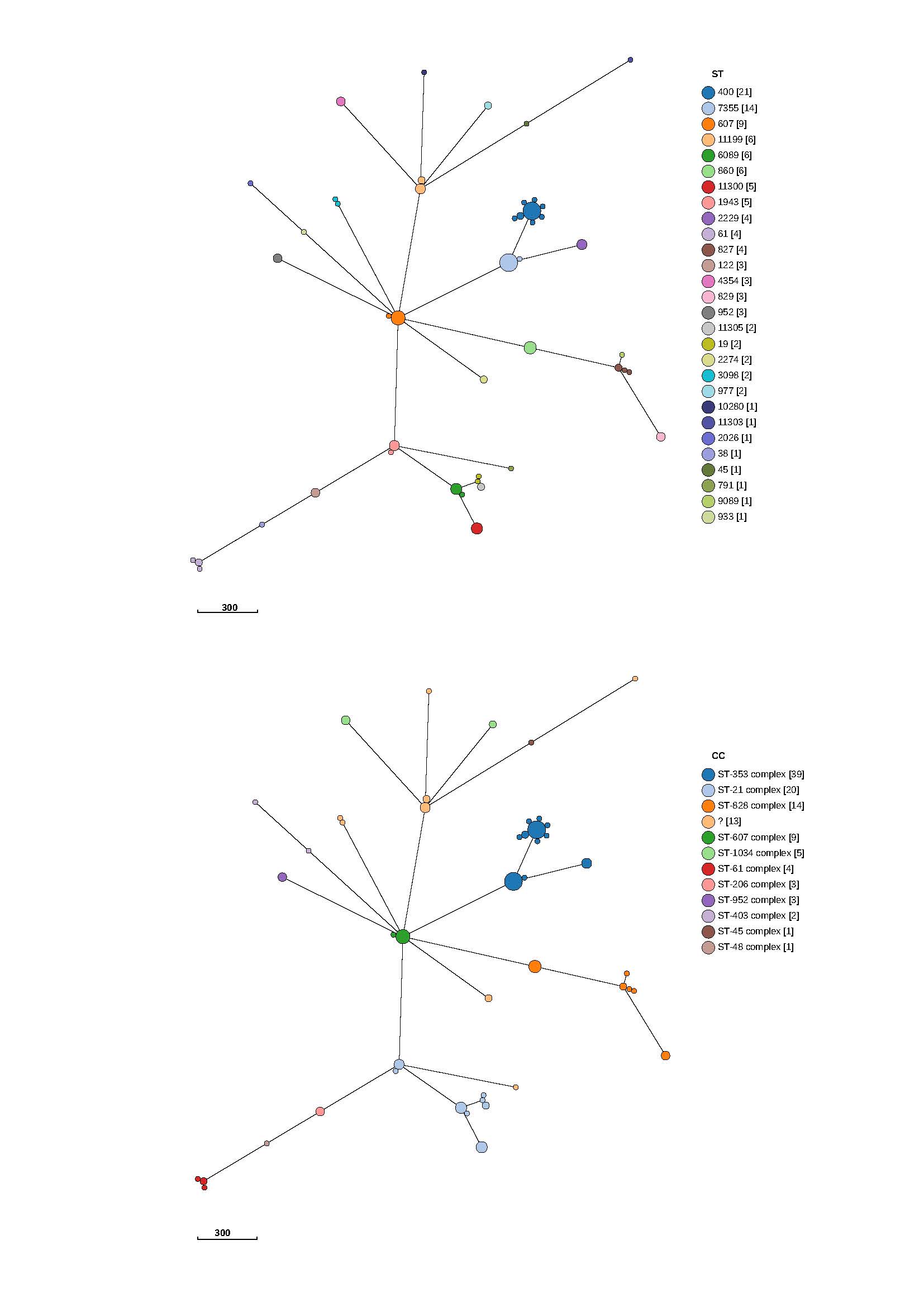


**C)**


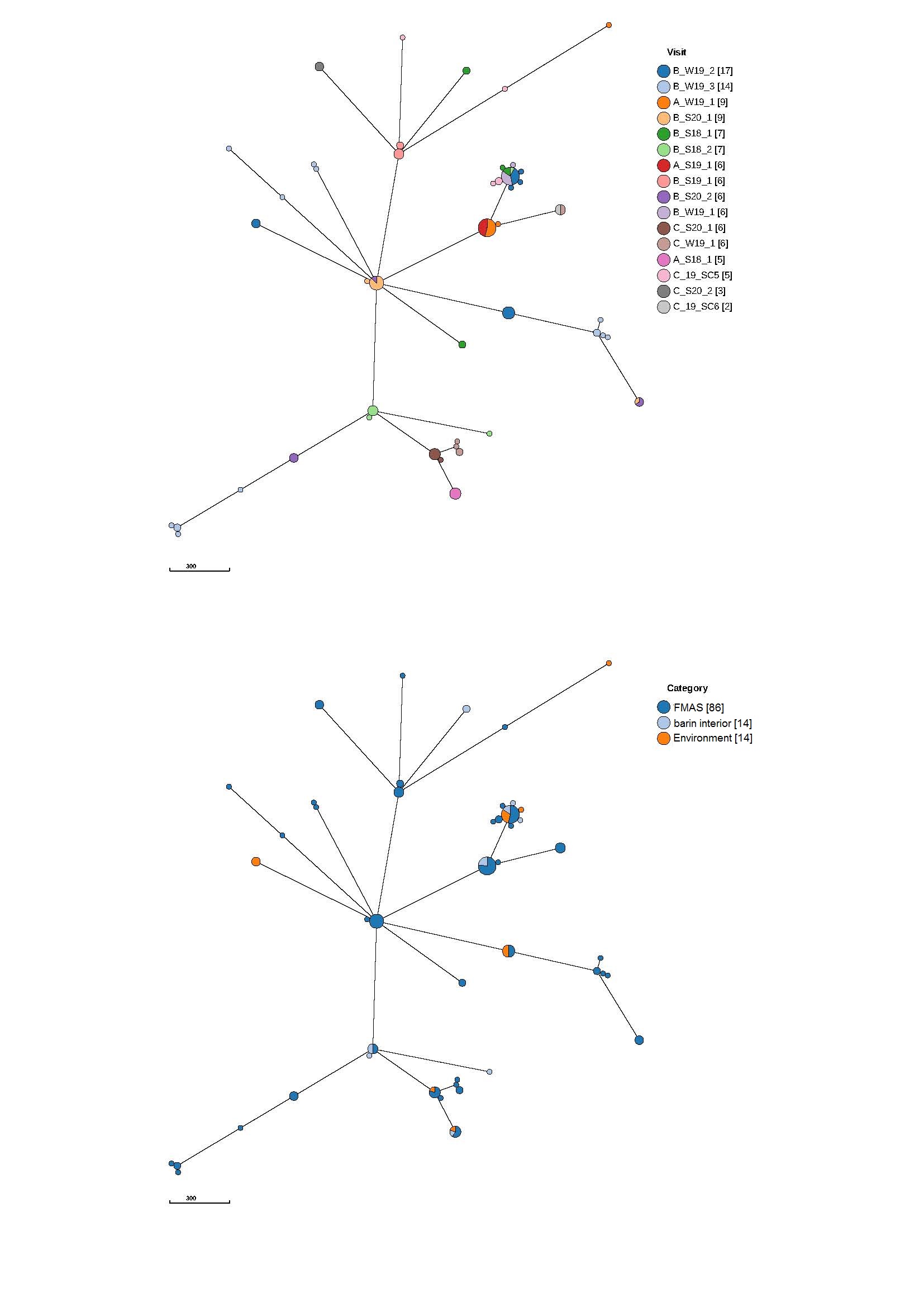


**Figure A-C):** Minimum spanning trees shows relationship between different MLSTs based on the 7 housekeeping genes colored by A) Farm and season, B) ST and CC and C) Visit and category.

**1)**


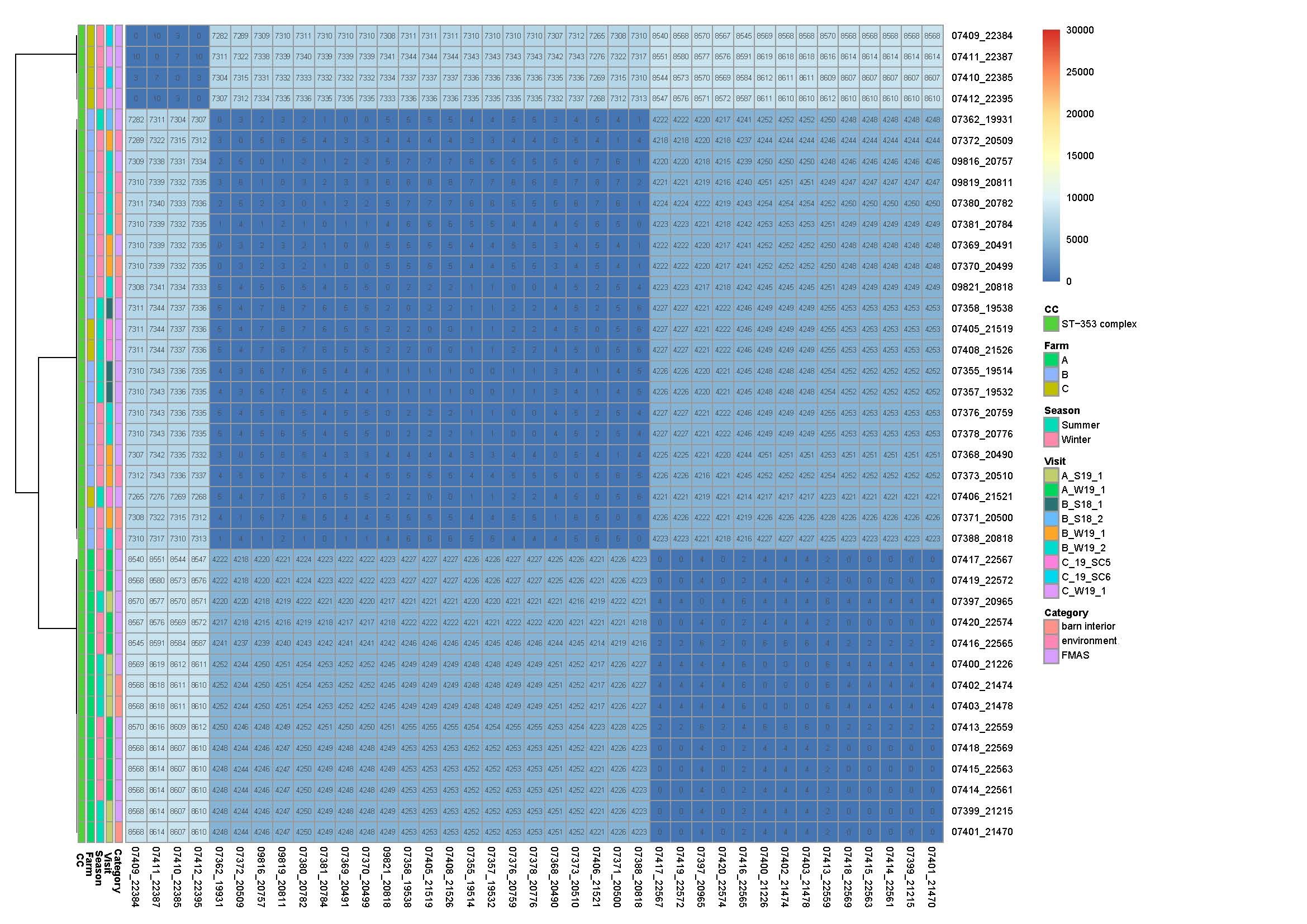


**2)**


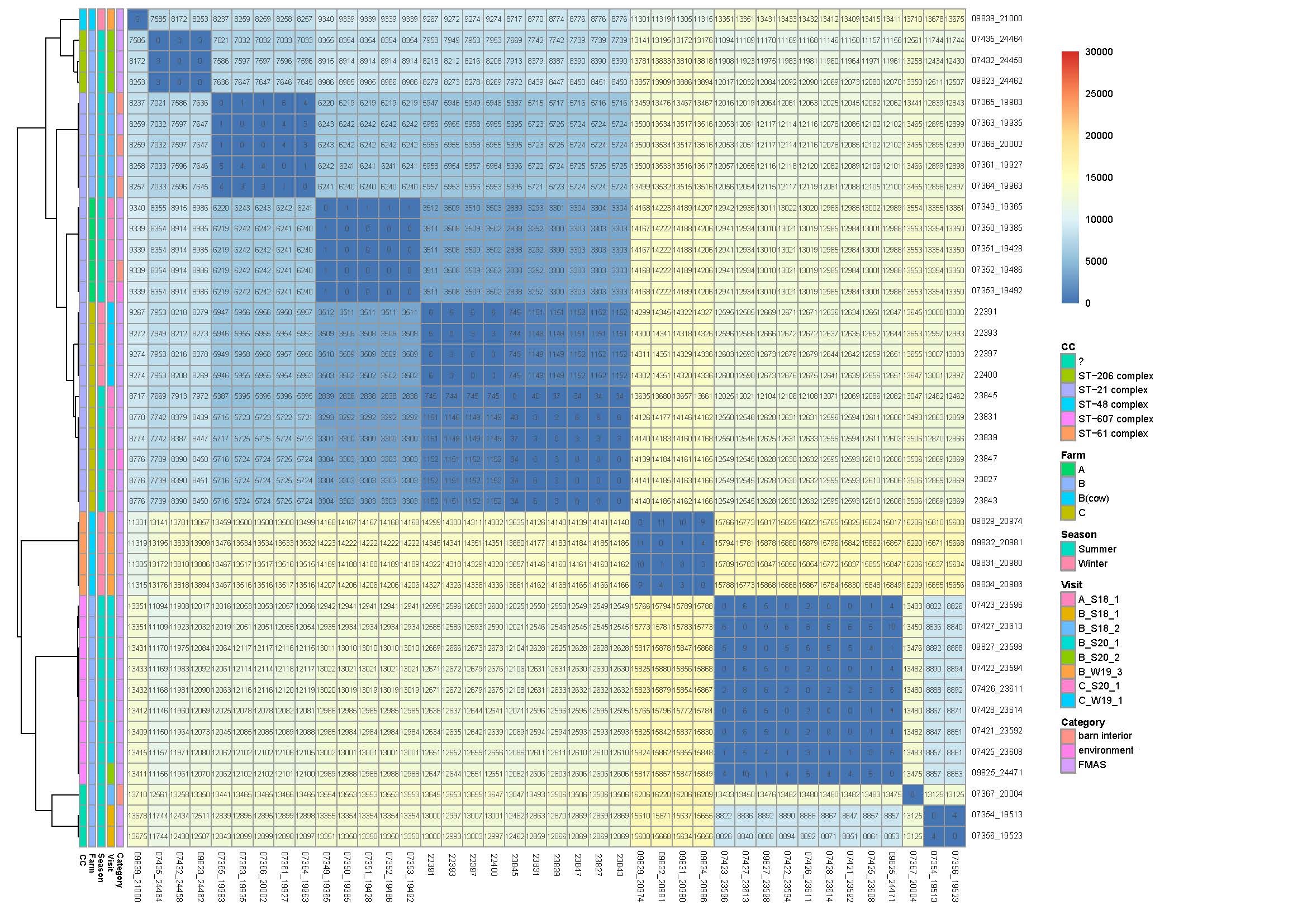


**3)**


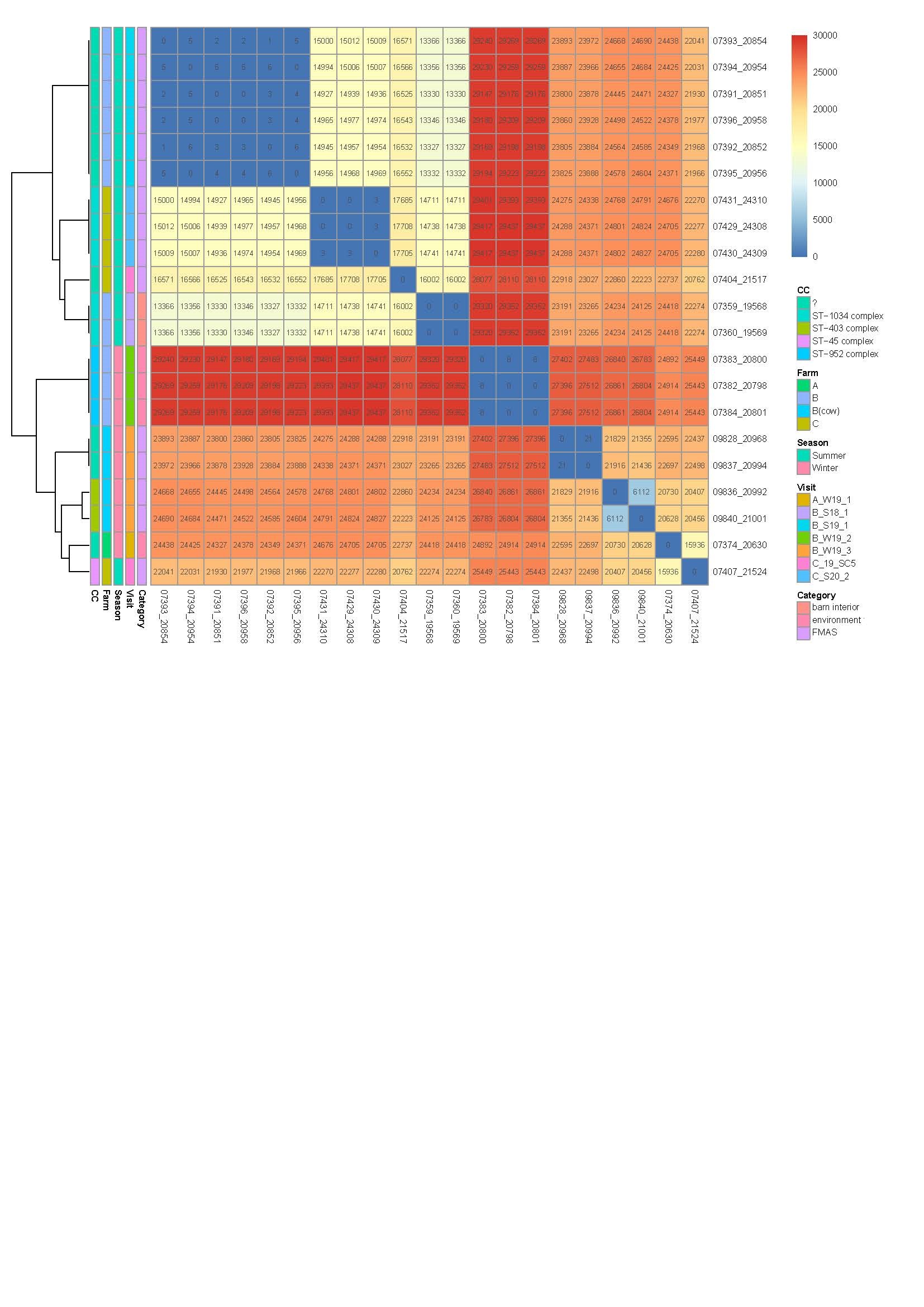

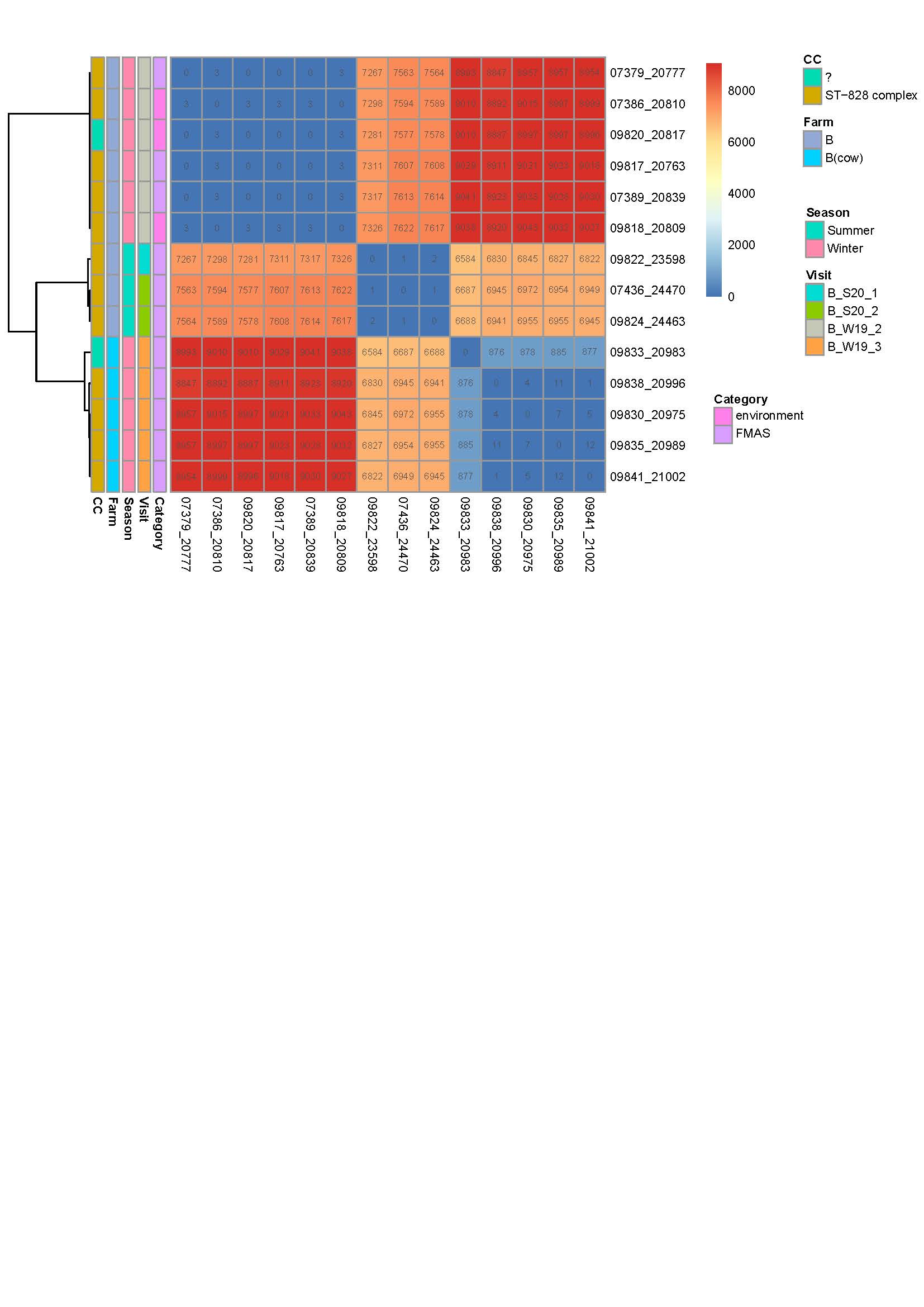


**Figure 1-4:** The single nucleotide polymorphism (SNP) distances of the gene sequence alignment of the core genome of *C. jejuni* (1-3*)* and *C. coili* (4) were calculated with snp-dists v.0.7.0.4 and visualized as heatmap by the R package pheatmap. Colours in the heat map represent the number of SNPs difference as shown in the right legend.

**MLST-ST types**

New multilocus sequence typing (MLST) alleles and MLST-ST types were uploaded to PubMLST.org/campylobacter.

| **id** | **isolate** | **country** | **continent** | **region** | **year** | **sender** | **aspA** | **glnA** | **gltA** | **glyA** | **pgm** | **tkt** | **uncA** | **ST (MLST)** | **clonal_complex (MLST)** |
| --- | --- | --- | --- | --- | --- | --- | --- | --- | --- | --- | --- | --- | --- | --- | --- |
| 110667 | 210616_21-07391_20851 | Germany | Europe |  | 2019 | 557 | 22 | 28 | 4 | 762 | 363 | 7 | 35 | 11199 |  |
| 110668 | 210616_21-07392_20852 | Germany | Europe |  | 2019 | 557 | 22 | 28 | 4 | 762 | 363 | 7 | 35 | 11199 |  |
| 110669 | 210616_21-07394_20954 | Germany | Europe |  | 2019 | 557 | 22 | 28 | 4 | 762 | 363 | 7 | 35 | 11199 |  |
| 110670 | 210616_21-07395_20956 | Germany | Europe |  | 2019 | 557 | 22 | 28 | 4 | 762 | 363 | 7 | 35 | 11199 |  |
| 110671 | 210616_21-07396_20958 | Germany | Europe |  | 2019 | 557 | 22 | 28 | 4 | 762 | 363 | 7 | 35 | 11199 |  |
| 110736 | 210616_21-07349_19365 | Germany | Europe |  | 2018 | 557 | 2 | 791 | 12 | 3 | 2 | 1 | 5 | 11300 | ST-21 complex |
| 110737 | 210616_21-07350_19385 | Germany | Europe |  | 2018 | 557 | 2 | 791 | 12 | 3 | 2 | 1 | 5 | 11300 | ST-21 complex |
| 110738 | 210616_21-07351_19428 | Germany | Europe |  | 2018 | 557 | 2 | 791 | 12 | 3 | 2 | 1 | 5 | 11300 | ST-21 complex |
| 110739 | 210616_21-07352_19486 | Germany | Europe |  | 2018 | 557 | 2 | 791 | 12 | 3 | 2 | 1 | 5 | 11300 | ST-21 complex |
| 110740 | 210616_21-07353_19492 | Germany | Europe |  | 2018 | 557 | 2 | 791 | 12 | 3 | 2 | 1 | 5 | 11300 | ST-21 complex |
| 110741 | 210616_21-07374_20630 | Germany | Europe |  | 2019 | 557 | 1 | 172 | 95 | 426 | 1128 | 353 | 6 | 11303 |  |
| 110742 | 22397 | Germany | Europe |  | 2018 | 557 | 2 | 1 | 5 | 887 | 2 | 1 | 5 | 11305 | ST-21 complex |
| 110743 | 22400 | Germany | Europe |  | 2019 | 557 | 2 | 1 | 5 | 887 | 2 | 1 | 5 | 11305 | ST-21 complex |
